# Implementing an Intervention for BPSD at Inpatient Mental Health: Feasibility & Acceptability

**DOI:** 10.1093/geroni/igaf122.3963

**Published:** 2025-12-31

**Authors:** Melissa Meynadasy, Kelly O’Malley, Holly Marston, Amanda Smith, Kelly Klein

**Affiliations:** VA Boston Healthcare System, West Roxbury, Massachusetts, United States; VA Boston Healthcare System, Boston, Massachusetts, United States; VA Boston Healthcare System, Brockton, Massachusetts, United States; VA Boston Healthcare System, Brockton, Massachusetts, United States; VA Boston Healthcare System, Brockton, Massachusetts, United States

## Abstract

Given population aging and increased dementia risk in substance use and serious mental illness populations, inpatient mental health (MH) will likely admit more older adults with comorbid dementia. Behavioral and psychiatric symptoms of dementia (BPSD) also result in admissions. Inpatient MH staff do not always receive training in BPSD. STAR-VA is an evidence-based intervention for BPSD developed for VA nursing homes, with demonstrated feasibility and efficacy in acute medical settings. Using the Institute for Healthcare Improvement model, this quality improvement project assessed feasibility and acceptability of STAR-VA in inpatient MH. Needs assessment examined interest and utility of dementia education; post-implementation assessment focused on acceptability and ongoing needs. Implemented changes focused on: education, staff support, collaboration, milieu. All respondents (N = 14; 78.6% nursing) reported caring for patients with dementia. Staff shortages (71.4%), BPSD (50%), lack of resources (50%), placement difficulty (42.9%), and burnout (42.9%) were most endorsed challenges. Only 28.6% of respondents agreed they had tools to improve the milieu. Educational needs included brief in-services (85.7%), dementia workshops (57.1%), and informal consultation (42.9%). Over 7 months, 17 patients were enrolled, 3 formal behavior plans were created, and nursing attendance at educational rounds ranged from 5-13 (M = 9). Follow-up questionnaires (N = 9; 77.8% nursing) revealed managing BPSD (66.7%) and burnout (55.6%) remained most challenging. Lack of resources decreased to 33.3% endorsed. Respondents agreed they had tools needed to improve the milieu (66.7%). Majority (88.9%) reported educational efforts were helpful for managing BPSD. It appears it is feasible and acceptable to adapt STAR-VA to inpatient MH.

